# The effect of yoga on hypertensive women’s blood pressure and sexual life: A randomized controlled trial

**DOI:** 10.12669/pjms.41.2.10656

**Published:** 2025-02

**Authors:** Gurcan Solmaz

**Affiliations:** 1 Dr. Gurcan Solmaz, Associate Professor, Istanbul University-Cerrahpasa Florence, Nightingale Faculty of Nursing, Istanbul, Turkey

**Keywords:** Hypertension, Sexuality, Randomized Controlled Trial, Yoga

## Abstract

**Objectives:**

To investigate the effects of a 12 weeks yoga program three times a week for one hour on blood pressure and sexual life among hypertensive women.

**Methods:**

This study was a pre-post test randomized controlled trial (NCT06319365) performed in accordance with the CONSORT guidelines. This study was conducted in a public hospital in Turkey between June 15, 2022 and October 30, 2022. Data were collected by blood pressure measurements, Personal Information Form and Arizona Sexual Experience Scale. The sessions of the yoga group were conducted online for one hour three days a week for 12 weeks. The control group did not receive any intervention during the study. No side effects were recorded throughout the study.

**Results:**

Participants in the yoga group had significantly lower systolic and diastolic blood pressure compared to the control group (p<0.001). In addition, women in the yoga group showed significant improvements in sexual activity, arousal, orgasm, lubrication and overall sexual function (p<0.05).

**Conclusion:**

A 12-week, three times a week yoga program reduced blood pressure and positively affected sexual functioning.

## INTRODUCTION

Factors such as hormonal changes, oral contraceptive use, obesity, and stress levels increase the predisposition to hypertension (HT) in women.^1^,^2^ Despite the use of numerous antihypertensive drugs, less than a third of women undergoing treatment achieve the targeted blood pressure. Therefore, different strategic approaches are needed to reduce blood pressure.^3^ Wolff (2016) shows that yoga can decrease systolic blood pressure (SBP) by up to 6 mmHg and diastolic blood pressure (DBP) by up to 5 mmHg. A 12-week yoga study conducted with a home video for women showed that DBP decreased according to the initial measurements.^4^ HT negatively affects the vascular system, sexual dysfunctions may occur.^5^

Although the number of studies on the relationship between HT and sexual function is limited, the issue has been primarily addressed as it relates to men’s sexual health. These vascular changes can cause sexual reluctance in women by negatively affecting the clitoral and vaginal vascular structures, leading to decreased blood flow in the pelvic region, thinning of the smooth muscles of the vaginal wall, vaginal dryness, and pain during the phases of sexual intercourse, orgasm, and arousal.^4^-^6^ In one of the few studies, it was determined that 42% of hypertensive women experience sexual dysfunction.^7^-^9^ Yoga is a practice that enhances physical and mental flexibility without side effects, aiding in health maintenance and improvement. When examining studies on changes in women who practice yoga, significant improvements were found in areas such as depression, body image, incontinence, and sexuality.^10^-^12^

In a study where a yoga program was applied to women with incontinence problems, it was found to reduce the associated inflammatory process, because most yoga asanas (postures) stretch the pelvic floor muscles, suggesting it could be applied as a complementary treatment. Asanas such as mula bandha (root lock) stretch the pelvic floor muscles, accelerate blood flow in the genital area, and improve sexual desire, pain, and sexual satisfaction.^12^,^13^ There is no study evaluating the effect of yoga on high blood pressure and sexual life of hypertensive women. Therefore, it is thought that this study will fill an important gap in the literature.

## METHODS

This study was conducted as a pre and post-test randomized controlled trial to determine the effect of online yoga practice for 12 weeks, three days a week for one hour, on blood pressure and sexual dysfunction in women with hypertension. It was conducted between June 15 and October 30, 2022 at the Cardiology Clinic of State Hospital in Turkey. The methodology was designed according to the CONSORT guidelines.

### Inclusion criteria:

- Women aged 18 years or older
- Diagnosis of hypertension for at least three months and currently receiving treatment
- Not pregnant
- Having the equipment (computer, smartphone or tablet) to participate in the online yoga program
- Willingness to participate in the research.

### Exclusion criteria:

- Illiteracy
- Unwillingness to participate in the research
- Having a severe mental disorder
- Having any condition, determined by an expert, that would hinder the practice of yoga.

The sample size was determined with reference to the study by Wolff, et al. (2016),^4^ at a significance level of p<0.05 and a confidence interval of 95%, it was determined that 80% power could be reached if a medium effect size of (0.5) was performed in independent groups with at least 25 people in each group. A total of 107 hypertensive women were included in the evaluation; women who did not meet the research criteria (n=22) and those who refused (n=15) were excluded. Hypertensive women who met the research criteria and agreed to participate in the study were given consecutive numbers and divided into groups according to their numbers. The 70 women who could participate in the study were assigned to the yoga (n=34) and control (n=36) groups with the Random.com computer program.

Since the researcher was solely responsible for the management of the yoga program, blinding could not be performed and the yoga practice was managed by the researcher in equal pairs. At the end of the study, the power of the study was calculated as 0.95 with α=0.05 and β=0.20. No side effects developed during the research process and no participant withdrew from the study. Data were collected twice, at the beginning of the study and at the end of the 12 weeks with an assessment at the hospital. Baseline data for both groups were collected at the hospital through one-on-one interviews. Blood pressure was measured with a mercury sphygmomanometer using the auscultatory method on the right arm while the participant was seated. The control group continued with their antihypertensive treatment, while the yoga group practiced yoga in addition to their antihypertensive treatment.

### Ethical considerations:

The study got approval from the University Human Research Ethics Committee (date March 22, 2022 number: 2022/33), and registered with the ClinicalTrials.gov number (NCT06319365). Throughout the process, adherence to the principles of the Helsinki Declaration was ensured.

### Data collection forms:

### Personal Information Form:

This Form included ten questions about the sociodemographic characteristics of the participants and their hypertension, based on similar studies and literature previously conducted by the researcher.^13^,^14^

### Arizona Sexual Experience Scale (ASEX):

This scale assessed sex drive, arousal, vaginal lubrication, ability to reach orgasm, and satisfaction from orgasm. The total score is the sum of the individual item scores, with a cut-off point of 11.^15^ Cronbach’s alpha coefficient was 0.90, indicating that the scale was valid for distinguishing sexual dysfunctions.

### Statistical analyses:

The data were analyzed using the Statistical Program- SPSS for Windows, 22.0 package program. Frequency analysis and descriptive statistics were performed to evaluate ASEX scores and blood pressure at pre- and post-test. The Shapiro-Wilk test was used to determine the non-parametric characteristics of the data. The Mann-Whitney U test was used for comparisons between groups, and the Wilcoxon signed-rank test was applied for within-group comparisons. All analyses were conducted at a 95% confidence level (p < 0.05).

## RESULTS

The mean ages of women in the yoga and control groups were similar (p>0.05). There was no significant difference between the groups in the pre-test blood pressures and ASEX scale comparisons (p>0.05). In contrast, a significant decrease of 9-11 mmHg was observed in the yoga group in the second measurements (p<0.05). In the control group, no change was recorded between blood pressure measurements (p>0.05). At the end of the study, there was a significant improvement in the sexual quality of life, drive, arousal, orgasm and sexual satisfaction values of the yoga group both within and between the groups (p<0.05). No change was recorded in the quality of sexual life of the control group between measurements (p>0.05).

**Table-I:** Pre and post-test blood pressures and ASEX scale comparisons in women.

Measurements		Yoga Group (n=35)	Control Group (n=35)	Difference (95% Confidence Interval)	p**
Age (years) X±SD		53.15±6.31	55.67±5.89	54.44±6.19	0.900
Sistolic Blood Pressure	1st	135.00±10.66	136.69±13.50	-1.69±2.84 (-7.56 to 4.17)	0.566
	2nd	113.47±7.05	132.66±18.23	-9.70±11.18 (-14.16 to -5.24)	<0.001
p*	<0.001	0.263		
Diastolic Blood Pressure	1st	82.24±10.44	82.53±17.43	-0.29±6.99 (-4.59 to 4.01)	0.893
	2nd	67.64±7.10	79.50±10.31	-11.85±3.21 (-16.10 to -7.60)	<0.001
p*	<0.001	0.209		
Arizona Sexual Experience Total	1st	21.23±4.11	22.25±3.40	-1.02±0.71 (-2.81 to 0.78)	0.263
	2nd	19.29±4.33	22.55±3.21	-3.26±1.12 (-4.79 to -1.15)	0.001
p*	0.010	0.070		
Drive	1st	4.02±0.79	4.16±0.69	-0.14±0.10 (-0.49 to 0.21)	0.445
	2nd	3.70±0.79	4.16±0.67	-0.46±0.12 (-0.82 to -0.09)	0.009
p*	0.014	0.324		
Arousal	1st	4.08±0.83	4.16±0.73	-0.08±0.10 (-0.45 to 0.29)	0.677
	2nd	3.70±0.75	4.16±0.77	-0.46±0.02 (-0.82 to -0.09)	0.015
p*	0.016	0.324		
Orgasm	1st	4.26±0.93	4.47±0.73	-0.21±0.20 (-0.60 to 0.19)	0.303
	2nd	3.70±0.87	4.58±0.69	-0.88±0.18 (-1.25 to -0.50)	<0.001
p*	0.001	0.601		
Sexual satisfaction	1st	4.17±0.90	4.36±0.76	-0.19±0.14 (-0.58 to 0.21)	0.358
	2nd	3.73±0.89	4.36±0.79	-0.63±0.10 (-1.03 to -0.22)	0.001
p*	0.005	0.160		
Satisfaction from orgasm	1st	4.67±1.09	5.08±0.93	-0.41±0.16 (-0.89 to 0.77)	0.090
	2nd	4.35±1.25	5.08±0.96	-0.73±0.29 (-1.26 to -0.19)	0.007
p*	0.216	0.324		

1st: At the initial data collection phase; 2nd: At the end of the third month; p*: Wilcoxon test; p**: Mann Whitney U-test.

## DISCUSSION

In this study, the effects of yoga practiced three days a week for three months on blood pressure and quality of sexual life in hypertensive women were evaluated. These findings are similar to other studies evaluating the effect of yoga on blood pressure.^16^-^18^ Yoga decreases sympathetic nervous system activity and increases parasympathetic nervous system activity. At this point, the level of nitric oxide in the blood increases and blood pressure can be stabilized by vasodilation. Furthermore, studies show that yoga lowers cortisol levels, a stress hormone associated with hypertension and stabilizes blood pressure by reducing stress.^17^-^19^ Considering that antihypertensive treatments reduce blood pressure by an average of 5-10 mmHg,^19^,^20^ it can be said that the significant reduction in blood pressure after three months with a yoga program three days a week is significant. The quality of sexual life of the women who participated in the study was low. Although there are studies showing that hypertension negatively affects sexual life in men, few studies have evaluated the effect of changes in blood pressure on the sexual life of women.^6^,^9^

This is the first study to include an experimental intervention to address sexual dysfunction in hypertensive women. With the three-month yoga program applied during the study, the yoga group showed a significant improvement in drive, arousal, orgasm, sexual satisfaction, orgasmic satisfaction and ASEX total scores from the first measurements to the follow-up (p<0.05). The moola bandhas, cat pose, and malasana movements in the yoga flow designed for this research are similar to Kegel exercises and contribute to the flexibility and strengthening of the pelvic muscles. Studies have found that these exercises reduce dysmenorrhea, menopausal symptoms, and sexual dysfunction problems.^13^,^14^,^21^,^22^ Other research evaluating the effects of yoga on female sexuality indicates that yoga increases sexual awareness, stimulates genital blood flow through its positive effect on peripheral blood flow, and strengthens pelvic floor muscles.^10^,^12^,^13^ All these data provide new information on the feasibility of applying yoga practices as a non-pharmacological approach for blood pressure and sexual health management in hypertensive women.

### Limitations:

This study provides evidence-based recommendations for accessible, low-cost, and sustainable home-based yoga programs that can improve blood pressure and sexual function in hypertensive women. The study’s limitations include a small sample size and a short three-month follow-up period.

## CONCLUSION

Study results indicate that a yoga program practiced three days a week for three months may help reduce blood pressure and positively influence specific aspects of sexual function, such as arousal, orgasm, and lubrication, in hypertensive women. To mitigate the negative effects of the physiological changes hypertensive women experience on blood pressure and sexual dysfunction, nurses can recommend simple yoga programs that can be practiced at home, three days a week for three months. Large-scale and long-term studies are needed to further validate these findings.
